# Out of their minds? Externalist challenges for using AI in forensic psychiatry

**DOI:** 10.3389/fpsyt.2023.1209862

**Published:** 2023-08-24

**Authors:** Georg Starke, Ambra D’Imperio, Marcello Ienca

**Affiliations:** ^1^Faculty of Medicine, Institute for History and Ethics of Medicine, Technical University of Munich, Munich, Germany; ^2^École Polytechnique Fédérale de Lausanne, College of Humanities, Lausanne, Switzerland; ^3^Munich School of Philosophy, Munich, Germany; ^4^Department of Psychiatry, Hôpitaux Universitaires de Genève, Geneva, Switzerland; ^5^Service of Forensic Psychiatry CURML, Geneva University Hospitals, Geneva, Switzerland

**Keywords:** artificial intelligence, machine learning, ethics, forensic psychiatry, social determinants of health

## Abstract

Harnessing the power of machine learning (ML) and other Artificial Intelligence (AI) techniques promises substantial improvements across forensic psychiatry, supposedly offering more objective evaluations and predictions. However, AI-based predictions about future violent behaviour and criminal recidivism pose ethical challenges that require careful deliberation due to their social and legal significance. In this paper, we shed light on these challenges by considering externalist accounts of psychiatric disorders which stress that the presentation and development of psychiatric disorders is intricately entangled with their outward environment and social circumstances. We argue that any use of predictive AI in forensic psychiatry should not be limited to neurobiology alone but must also consider social and environmental factors. This thesis has practical implications for the design of predictive AI systems, especially regarding the collection and processing of training data, the selection of ML methods, and the determination of their explainability requirements.

## The promises of AI-based precision psychiatry

Artificial Intelligence (AI) techniques, especially those based on machine learning (ML), are becoming integral part of procedures across medicine. Medical areas that can benefit from image classification enabled by computer vision, such as dermatology, radiology, pathology, or ophthalmology, provide ample examples for this development (1). Other domains of medicine are increasingly following suit though, and psychiatry is no exception. Here, ML-based models show a potential route to analyse complex multiscalar and multimodal data in a novel way, offering a way towards what has been called “precision psychiatry” (2).

As part of the broader move towards personalized medicine, precision psychiatry promises to enable healthcare strategies based on AI predictions and tailored more closely to individual patients. Clinical examples range from diagnostic and prognostic tools to improved opportunities for monitoring and treating psychiatric conditions (3). Identifying individual clinical phenotypes in bipolar disorders (4), predicting psychotic episodes in at-risk patients (5), managing mood disorders through using digital phenotyping (6) or selecting the most suitable psychopharmacological intervention in depression or schizophrenia (7–9) can seemingly all be improved by harnessing the computational power of ML for large-scale datasets. Ultimately, even the very classification of psychiatric disorders may be overhauled or at least refined by drawing on results from AI-based research (10–12).

Despite these large promises, there are important ethical concerns with applying AI in psychiatry (13). As examples from other medical domains have shown, embedding AI in clinical care can jeopardize patients’ safety if the AI has not been tested and validated rigorously in the correct context, resulting in potentially dangerous treatment recommendations (14). A study among US psychiatrists using different case vignettes showed that interacting with correct ML-based treatment recommendations did not improve physicians’ accuracy while incorrect treatment recommendations paired with persuasive explanations even decreased physicians’ accuracy of choosing a suitable psychopharmacological treatment (15). Such findings highlight the intricacies of involving AI in clinical decision-making processes and how overreliance on imperfect ML tools may adversely affect supposedly autonomous choices made by clinicians.

In line with the wider literature on AI ethics, particular attention has also been devoted to questions of fairness and bias (16). AI systems are known to be susceptible to existing social biases, which they potentially entrench and amplify. For instance, commercial gender classification systems for facial analysis have been shown to systematically perform worse on images of female and darker-skinned persons, with the worst classificatory accuracy for the intersectional group of darker-skinned females (17). In clinical contexts, addressing biases is particularly intricate due to the manifold biological, social, psychological and cultural factors influencing health and their often unclear causal interactions (18). For instance, a recent study on an AI-based decision support system in the treatment of heart failure in the US highlighted how racial biases may not be apparent in a system’s evaluation: the AI correctly predicted historical real-life treatment outcomes, yet these outcomes were themselves the result of a racially biased healthcare system (19). It may therefore sometimes be necessary to carefully curate training data and restrain optimization processes to achieve a less accurate but potentially more just model (19).

In light of this background, there are justified ethical concerns with expanding psychiatric predictions on a population level, as ML models with their well-documented propensity of reinforcing existing biases from the training data may provide many false positive predictions in specific disadvantaged communities. This would create self-fulfilling prophecies and further worsen discriminatory practices (20, 21). As we will see, such dangers become even more worrisome in the context of forensic psychiatry.

## Using AI for predictions in forensic psychiatry

To our knowledge no AI-based tool has as of yet entered routine use in forensic psychiatry. However, several approaches have been suggested through proof-of-concept studies. These attempts of AI-based neuroprediction, i.e., prediction of health or behavioural outcomes based on neurobiological factors utilizing AI, can be seen as part of a long-standing search for neuro-markers that supposedly render risk assessment more objective and specific. Already in the early 2000s, researchers attempted to determine the value of specific variables to predict the restorability of criminal defendants using regression analysis (22), or drew on functional neuroimaging and multi-voxel pattern analysis (MVPA) to gain insights into defendants’ thoughts (23). More recently, approaches within forensic psychiatry have integrated ML to assess psychopathy or make predictions about future aggressive behaviour. Training approaches as well as training data differ largely across such studies. A recent Danish study for instance predicted criminal offenses during or after psychiatric care using sociodemographic information, psychiatric history and criminal history as training data (24). In a similar approach, a Swiss team used machine learning to explore a large set of comprehensive information including forensic patients’ psychiatric and criminal history, socio-demographic and prison data, social and sexual functioning, childhood experiences to identify variables that best predict aggression in patients with schizophrenia (25). Other research avenues have focused primarily on employing ML on neural data. A meta-analysis by Deming and Koenigs for instance analysed findings from 25 original studies employing functional MRI to identify functional neural correlates of psychopathy, which in turn is related to future criminal offenses (26).

Previous research has already raised ethical concerns about risk assessments of violence in forensic psychiatry, with and without the assistance of AI. For instance, in their ethical treatment of tools assessing risk of violence with structured questionnaires, Douglas and colleagues identified overreliance on the resulting scores, mismatches between applications and contexts, risks of discrimination and stigmatization, and the premature exclusion of contentious demographic variables as main concerns (27). These concerns are mirrored in the relatively scarce ethical literature dedicated specifically to AI in forensic psychiatry. Richard Cockerill for instance has drawn on the four principles of biomedical ethics by Beauchamp & Childress (28) to map and discuss ethical challenges posed by ML-based predictions of future violent behaviour with view to non-maleficence, beneficence, respect for autonomy and justice. Adding a neurolaw perspective to the debate about AI in forensic psychiatry, Tortora and colleagues have called for more research into the risks and benefits of neuroprediction as the technology matures (29).

In this paper, we approach the ethical debates surrounding the use of AI in forensic psychiatry from a complementary angle, expanding on the challenges that arise when employing AI in this field. We argue that, in addition to the many warranted ethical worries with AI in psychiatry and forensic psychiatry in particular, the very conceptualization of psychiatric disorders poses problems that have not yet received sufficient attention. In particular, we highlight that considering the external conditions that contribute to psychiatric disorders, rather than focusing exclusively on neural data, has practical implications for designing AI systems in forensic psychiatry. To do so, we first discuss the motivation for looking for AI-based tools by highlighting the unsatisfying status of current assessment practices. We then turn to the recent literature on the conceptualization of psychiatric disorders and highlight empirical and theoretical arguments supporting an externalist stance, i.e., the position that what goes on in a (disordered) mind cannot solely be explained by reference to individual bodily and neural processes (30–33). We then spell out the implications of these insights for ongoing research on AI-based tools in forensic psychiatry and provide four practical recommendations how to move forward.

## A problematic status quo

A standard strategy to evaluate AI-based systems in medicine is to benchmark them against the current state of the art in clinical practice (34). When discussing potential ethical pitfalls of predictive AI in forensic psychiatry, it is therefore important to understand the current status quo of assessment practices in forensic psychiatry and their own potential ethical shortfalls, to have a clear point of comparison (27). In addition, being aware of existing problems in forensic evaluations may also foster a better understanding why many researchers are motivated to explore AI-based solution in forensic psychiatry, in the hopes of improved tools.

Current practice in forensic psychiatry is commonly supported by structured scales which are used to evaluate defendants and support professional recommendations in court. As there are large differences in the practice of forensic psychiatry worldwide, not least owing to different legal traditions (35), forensic practice in Switzerland may serve as an example here. Here, the prevailing practice involves subjecting a single defendant to evaluation by two distinct experts concurrently. These two experts are obligated to individually conduct interviews, each lasting approximately 60 min, during which they gather the defendant’s comprehensive medical history and consider information provided by other medical professionals who may have been involved in the defendant’s case. This dual assessment consitutes an important step to mitigate potential interpretational biases (36). To further foster impartiality, different psychometric scales are utilized. While the use of scales is not mandatory, they support a more objective assessment of the risk and responsibility associated with the defendant’s actions, thereby facilitating a clearer presentation of evidence in the courtroom (36). Given that AI-based recommendations would likely play a role similar to such scales, it is important to understand their use and limitations. A prominent example, the Hare Psychopathy Checklist-Revised (PCL-R) (37) can serve as a useful point of comparison.

The PCL-R is used to distinguish between narcissistic and antisocial traits. It contains items such as shallow affects, superficial charm, and pathological lying, which are ranked on a scale from 0 to 2. Originally, the scale was based on a single psychiatric report, reflecting the degree of resemblance between the assessed individuum and a prototypical psychopath examined by Robert Hare in 1980 (38). The scale was later revised based on a larger study, yet drew exclusively on male prisoners in Northern America (39). Despite this origin, PCL-R is one of the most frequently used scales in forensic psychiatry, both in court and research (40, 41). Since the assessment relies on the judgment of individual assessors it can suffer from interpretability bias and is prone to influence by defendants unless the assessing expert is sensitive to a potential manipulative attitude of the assessed person. Accordingly, research suggests that the scale is unreliable, offers incorrect and harmful conclusions, and is prone to misuse in legal systems (42). A training course for the use of the PCL-R aimed at strengthening the skills of the forensic psychiatrist exists (43). However, the scales are to be considered only as a tool used at the discretion of the expert. Moreover, in a hypothetical view, we might also think a forensic expert may evaluate an NGIR (Not Guilty for Insanity Reason) condition because of its susceptibility to the manipulative and narcissistic defensiveness of the evaluee.

Problems with scales used in forensic psychiatry are not limited to the PCL-R though. A study investigating the precision of two so-called actuarial risk assessment instruments (ARAIs), namely the *Violence Risk Appraisal Guide* (VRAG; 44) and the *Static-99* (45), found that both instruments, designed to predict future violent behaviour, entailed so much statistical uncertainty on the individual level “as to render [their] risk estimates virtually meaningless (46).”

Despite all these shortfalls, forensic psychiatrists have to make judgements when called upon to assess the risk and dangerousness of pathological behaviour (e.g., determining the risk of violent recidivism in persons accused of murder). While the individual risk of recidivism remains shrouded in uncertainty, a medico-legal compromise must be reached in court. Many well-known cases confirm how delicate a balance must be struck in forensic evaluation, and how large the stakes are, for individual defendants as much as for society. A notorious example from Italy is the so-called “Circeo Massacre.” In 1974, a year prior to the massacre, Mr. Angelo Izzo, one of the three perpetrators, was granted semi-release by a probation court after having been arrested for raping two women. This decision was made on the basis of his perceived “good behaviour” (47). Izzo served in jail only 10 months. Shortly after his sentence was suspended, he became one of the perpetrators of kidnapping and raping two young women, one of whom died. After serving approximately 25 years for the massacre (briefly interrupted by an escape to France in 1993), Izzo was granted in 2004 semi-freedom from Campobasso Prison based again on good behaviour in order to work at a cooperative called Città Futura (Future City). Nine months later, he murdered again two women (a 49 years old woman and her 14-year-old daughter). In opposition to such attention grabbing *false negative* cases, when defendants were falsely assessed to be unlikely to reoffend, there is also the danger of misclassifying defendants as high-risk, even though they do not pose a danger to society. Unfortunately, this may happen rather frequently. A systematic review and meta-analysis of 68 independent studies, including data from 24,847 persons from 13 countries, found that while the nine most frequently used assessment tools for risk of violence, sexual, and criminal behaviour had relatively high negative predictive value (median accuracy 91%), their positive predictive values were low to moderate (median accuracy 41%) (48). The authors therefore concluded that “even after 30 years of development, the view that violence, sexual, or criminal risk can be predicted in most cases is not evidence based” (48).

Given this unsatisfactory state of affairs, it is of little surprise that forensic psychiatry has turned towards machine learning to tackle complexity and provide better and more accurate predictive tools. Yet, also this approach is fraught with challenges and has to circumnavigate particular conceptual shallows if it is to move the debate forward. One key question researchers need to consider is what type of data should be included in the training of their models.

## Locating mental disorders: the challenge of externalism

There is intense ongoing debate in the philosophy of psychiatry as to the nature of psychiatric disorders and how to properly conceptualize psychiatric diagnoses. Our aim here is not to weigh in on long-standing disagreements supported by a rich academic tradition (49–51) but to point towards specific implications of recent scholarship for employing AI in forensic psychiatry.

One widely held position among biologically oriented psychiatrists looks at mental disorders primarily as brain disorders (52). The development of the research domain criteria (RDoC), spearheaded by the US NIMH, aiming for a diagnostic classification based on biological differences instead of symptoms, constitutes a prominent example for such a line of reasoning (53). This approach, which is also rather common among proponents of computational psychiatry (54), therefore locates the psychiatric problem that requires evaluation and treatment *within* the patient or defendant themselves. This position is increasingly called into question though by theories of mental disorders that one may call externalist (49, 55–58). As Roberts and colleagues summarize, such positions “hold that a comprehensive understanding of mental disorder cannot be achieved unless we attend to factors that lie outside of the head: neural explanations alone will not fully capture the complex dependencies that exist between an individual’s psychiatric condition and her social, cultural, and material environment (57).”

Embracing an externalist view does not entail rejecting the idea that psychiatric disorders are brain disorders. Rather, externalist theories emphasize the importance of looking beyond the brain in order to fully understand these disorders. In this regard, they are related to philosophical accounts that analyse mental processes as situated, embodied, embedded, enacted and extended within a specific extra-cranial environment (49, 59). For our argument, two points concerning the development and sustention of psychiatric disorders are particularly pertinent.

Ample empirical evidence highlights the etiological importance of environmental factors with view to the development of psychiatric disorders (60). Biological factors that are implicated in psychopathological aetiology are frequently linked to sociodemographic inequalities, such as a history of migration, living circumstances in urban areas, childhood adversity, or cannabis use (61). Schizophrenia with its many known individual genetic factors (62) is a case in point. While the heritability of schizophrenia is estimated to fall in the range of 41–87% (63), developmental factors heavily shaped by the respective environment play a key role for gene expression and co-determine whether an inherited genetic risk leads to schizophrenia in individual patients (64). Individual biological risks therefore constitute only one important factor in a complex, multifactorial aetiology.

At the same time, the individual expression and sustention of psychiatric disorders are similarly intertwined with an individual’s social environment. Arguing for an ecological view of the human brain, Fuchs calls this circular causality (55, 65): social feedback loops contribute to eliciting and sustaining dysfunctional states, such as unrequited stress reaction, which in turn again influence the social environment. Empirically, such interactions can be traced in the rich field of social neuroscience, looking at brain processes during reciprocal social interactions (66). Given that social-cognitive skills are intricately intertwined with the ability to make moral decision (67), social external factors are especially relevant to consider in forensic psychiatry: the possible presence of a responsible third party could alleviate or aggravate the sentence of a convicted person, for whom a psychiatric expertise is required to determine his criminal responsibility.

A full account of a forensically relevant mental disorder therefore needs to look closely at social influences, for “what goes on inside the head cannot be isolated from an organism’s interaction with the world (58).” This becomes especially clear when considering the expanding field of neuroscience that highlights the impact of poverty and social inequalities on cortical and subcortical brain structure as well as on brain function, affecting circuits that are implicated in language, emotion processing, memory, and executive functioning (68). Given that poverty seems to already affect brain function in infants (69) and has a lasting impact on the developing brains of children and adolescents (70), even a psychiatric diagnosis based purely on neurobiology may well reflect social inequalities. Adding a potentially opaque AI techniques to this complex causal mesh risks to further reify and amplify such existing inequalities.

## Consequences for potential AI applications in forensic psychiatry

An externalist view of psychiatric disorders has important implications for using AI in forensic psychiatry. If mental illnesses are indeed “inseparable from the patient’s lifeworld or social environment” (55), this should impact the selection of training data, the selection of appropriate models, the interplay between trained psychiatrists and AI models, and educative needs.

First, with view to selection of training data, researchers should always include social and environmental aspects in their data and go beyond, e.g., purely brain-based predictors of violent behaviour. Such data may include information about family and friendship networks, employment, income, place of residence, housing situation, and life events. Without controlling for such factors, there is a grave risk of turning social problems into supposedly psychiatric ones. At first, this may seem counterintuitive since an exclusive focus on biological data seems less prone to human bias. However, if the social and the biological dimension of the phenomenon cannot be disentangled, excluding environmental factors would not make AI less biased but rather render models blind to important mediating factors. Instead, developers should include such environmental and social determinants of (mental) health and actively scrutinize their data and models with view to potential sources of biases (71).

Second, researchers should prefer dimensional and dynamic models over categorical and static assessments. It has rightly been argued that, as clinical utility of AI models in psychiatry increases, so does their complexity (72). Nevertheless, research should still aim for simplicity were feasible and for conceptualizing a standardized and high-quality system, to avoid creating a complex algorithm that only calculates the error of the weighting error and moves away from the goal of the research. A dynamic application of variables, in which items are intended to evolve over time, could aim at predicting the treatability of the defendant, including environmental protective factors in the risk assessment. In this sense, AI could mirror existing scales such as the SAPROF (Structured Assessment of Protective Factors for violence risk), which considers potential reintegration as a distinctive feature (73).

A third point concerns the interaction between human practitioners and predictive AI models. One of the most important goals of forensic psychiatry is not only to assess a particular diagnosis of the evaluee, but also to evaluate the pre-existing dialectic between a psychiatric diagnosis, if any, and the crimes charged. It follows from this that the forensic psychiatric evaluation is aimed at a deep psychopathology investigation, often considering details that may be overlooked in simpler psychiatric assessments. To preserve this benefit, AI systems in medicine should therefore not replace physicians, as recent ethical guidelines have stressed again (74), but merely assist them in their practice. In addition, minimal demands of explicability and contestability, which are of general importance in medical AI, need to be respected, *especially* in a context such as forensic psychiatry where freedom is restricted. Consequently, one important goal for AI in forensic psychiatry would be the development of a personalized rehabilitation strategy that considers not only the diagnosis of the person being evaluated but also all the multifactorial factors, including cultural ones, involved in their unique lifeworld.

Finally, using AI in forensic psychiatry will require extensive education of all parties who rely on its recommendations, both from the medical as well as from the legal field. The earlier example of a complex interplay between genetics and environment in psychiatric disorders can be seen as paradigmatic here, raising similar critical issues with view to prediction (75). Ethical concerns have been raised that psychiatrists and genetic counselors may at times not fully understand the procedure and implication of psychiatric genetic testing, and require further training before using them in a beneficial manner (76). Potential knowledge gaps are even more concerning when it comes to the responsibility of predicting future criminal behaviour. As has been suggested, genetics can take on a dual role here: it can either serve to exculpate a defendant, who is subject to the unstoppable force of their genes, or it can be used to fuel essentialist intuitions that a supposedly objective test tell us something fundamental about a person’s very core (77). Employing AI in psychiatry should avoid both pitfalls, for which additional education remains crucial (78). Table 1 provides an overview of these points to consider and their associated normative implications.

**Table 1:** Overview of points to consider and their associated normative implications.

Points to consider	Normative implications
Selection of training data	Environmental aspects should be included in training data to avoid turning social problems into psychiatric ones. Excluding environmental factors may render researchers blind to important mediating factors.
Models to be preferred	Dimensional and dynamic models should be preferred over categorical and static assessments. A dynamic application of variables could aim at predicting the treatability of the defendant, including environmental protective factors in the risk assessment.
Interaction between human practitioners and AI	AI systems should not replace physicians but assist them in their practice. Explicability and contestability are important in AI systems, especially in forensic psychiatry where freedom is restricted. A personalized rehabilitation strategy should be developed that considers all the multifactorial factors involved in the defendant’s unique lifeworld.
Education of all parties	Potential knowledge gaps need to be addressed through extensive education of all parties who rely on AI recommendations. Additional education remains crucial to avoid the pitfalls of genetic essentialism and to make sure that AI is employed in a beneficial manner.

## Conclusion

In conclusion, we argue that any potential predictive AI system in forensic psychiatry must take into account the influence of social and environmental factors on the presentation and development of psychiatric disorders. Adopting such an externalist perspective on mental disorders has critical implications for the design and implementation of AI systems in forensic psychiatry. By emphasizing the need to consider the external environment, such as social and environmental factors, in the selection of training data and machine learning models, AI systems can avoid the risk of turning social problems into psychiatric ones and better account for important mediating factors. Additionally, the use of dimensional and dynamic models, human-machine interaction, and personalized rehabilitation strategies can help to improve the precision and humaneness of forensic psychiatry practices. However, these developments should be accompanied by extensive education for all parties involved to address potential knowledge gaps and ethical concerns, especially when it comes to predicting future criminal behaviour. Overall, our paper emphasizes the importance of responsible and ethical development of AI systems in forensic psychiatry that aim for better assessment and treatment. Yet, until these points have been addressed, doing justice to the complex interaction of social, mental and biological factors, forensic psychiatrists should not rely uncritically on predictive AI techniques, to avoid unintended consequences and negative societal impact.

## Author contributions

All authors listed have made a substantial, direct, and intellectual contribution to the work and approved it for publication.

## Funding

This work has been supported by the ERA-NET NEURON project HYBRIDMIND (Swiss National Science Foundation 32NE30_199436).

## Conflict of interest

The authors declare that the research was conducted in the absence of any commercial or financial relationships that could be construed as a potential conflict of interest.

## Publisher’s note

All claims expressed in this article are solely those of the authors and do not necessarily represent those of their affiliated organizations, or those of the publisher, the editors and the reviewers. Any product that may be evaluated in this article, or claim that may be made by its manufacturer, is not guaranteed or endorsed by the publisher.
